# Reinfection and reactivation of SARS-CoV-2

**DOI:** 10.2217/fvl-2021-0212

**Published:** 2022-09-26

**Authors:** Razieh Dowran, Amirmasoud Rayati Damavandi, Talat Mokhtari Azad

**Affiliations:** ^1^Department of Virology, School of Public Health, Tehran University of Medical Sciences, Tehran, Iran; ^2^Student Scientific Association of Virology, Student Scientific Research Center, Tehran University of Medical Sciences, Tehran, Iran

**Keywords:** COVID-19, reactivation, reinfection, SARS-CoV-2

## Abstract

As the cases of SARS-CoV-2 infection escalates, the essence of in-depth knowledge around acquired immunity and emergence of reinfection and reactivation have to be captured. While being a rare phenomenon, reinfection occurs as the result of diminishing protection conferred by antibodies, especially IgG. Reactivation is more concerned with the role of various elements including shedding lingering viral RNA for a prolonged time and incomplete resolution of infection along with the insight of dormant viral exosomes’ role. The concept of testing positive after two consecutive negative results requires proper discrimination of reinfection from reactivation. In this review, we summarized the current evidence for possible mechanisms leading to viral reactivation or test re-positivity. We also pointed out risk factors associated with both reinfection and reactivation.

The novel coronavirus reported from Wuhan city, China in December 2019 [1], has made scientists and healthcare policy makers reconsider their evaluations of the coronavirus family while accounting for more than 163 million cases with over 3 million deaths all around the globe as of 19 May 2021 [2,3]. The coronavirus family are among viruses capable of establishing respiratory infections. This family is comprised of several human pathogens including HCoV 229E, HCoV OC43, HCoV NL63 and HCoV HKU1 causing mild respiratory infection, SARS-CoV and the Middle East Respiratory Syndrome Coronavirus (MERS-CoV) famous for their pneumonia outbreaks and fatalities in China and Saudi Arabia, 2002 and 2012, respectively [4,5], and the novel coronavirus of SARS-CoV-2 with the ongoing pandemic [6]. In endemic areas for seasonal human coronaviruses of 229E, OC43 and NL63, starting new infections following primary one is reported to be common [7]. This may partly be because of their waning immunity by a decline in antibodies level in the course of over 6 months and significantly at 12 months after infection [8]. However, this is not the case for SARS-CoV and MERS-CoV protection due to their longer-lasting antibodies up to 24 and 34 months, respectively [9–12]. Even one study pointed out the durability of MERS-CoV neutralizing antibodies as long as 6 years after infection [13]. While seeking the shreds of evidence of immunity dynamics of SARS-CoV-2, we will discuss reinfection and also the resurgence of viral infection with possible mechanisms and risk factors in addition to feasible predictive markers for evaluating further cases.

## Criteria for true reinfection & reactivation of the virus

To elucidate the distinction between these two concepts some criteria can be applied to distinguish patients with various test results and clinical symptoms. In terms of reinfection, samples taken from patients must be sequenced genetically to identify a different clade rather than the first infection, confirmed true infection using real-time RT-PCR at each episode with high viral load (C_t_ <35), two consecutive negative results by RT-PCR at the episodes interval, immune responses dissimilarities in two episodes, a history of exposure to suspected individuals with COVID-19 and the time frame of more than 60 days after the first episode of infection is essential [14–17]. On the other hand, reactivation or relapse takes place mainly after recovering from an infection, the patient retests positive with the same viral clade or develops clinical symptoms no longer than 60 days and mainly occurred 10–27 days after infection added by lack of exposure with infected ones or exposure to infected ones with the virus from different clade [15,17,18]. In general, reactivation is more common in immunosuppressed patients [19]. This may be because of an unresolved viral infection that was not detected after two negative RT-PCR tests. The false negative RT-PCR test at the time of symptom resolution in the first episode is highly dependent on the technique used to retrieve samples, viral load and the site of the sampling [20]. Each of these concepts has different mechanisms and risk factors that turn individuals prone to either reinfection or reactivation.

## Reinfection

### Review of cases

For assessing the possibility of reinfection in non humans, one animal model study for SARS-CoV-2 reinfection displayed no signs of infection, viral shedding or clinical manifestation in rhesus macaques monkeys after second exposure with the same strain of the virus in first infection [21]. This might imply that SARS-CoV-2 prior exposure in rhesus macaques could confer immunity against possible future infections; however, human immunity is further in doubt due to several cases of reinfection reported around the globe.

The first case report of confirmed reinfection came out from Hong Kong that described a 33-year-old man with the typical symptoms of infection including cough, fever and sore throat three days before testing positive on 26 March 2020. Following being under observation at the hospital he was discharged on 15 April 2020, and two RT-PCR tests resulted in negative after symptom resolution. However, on the screening performed at the Hong Kong airport on 15 August 2020, he was found positive for infection but in this episode he was asymptomatic. The interval between the two episodes was 142 days and whole genome sequencing showed that the viral clade in the initial infection belonged to the clade of B.2 and for the second one B.1.79 with several nucleotide differences. These data are in line with the report of a patient who travelled to Europe with a similar circulating variant prior to the second infection. Moreover, the features of infection in the two episodes differed to some extent. Serum SARS-CoV-2 IgG analysis 10 days after the first infection and also 1 day after the second episode was reported to be negative; however, following 5 days of the second episode the results turned positive. Also, the higher viral load and CRP levels and amino acid changes in B-cell and T-cell epitopes in the second episode may imply different immune responses against distinct variant [22].

Another case of confirmed reinfection occurred in the US state of Nevada. In the report, a 25-year-old male was detected positive on 18 April 2020, while bearing the symptoms of infection which had begun on 25 March 2020. After a symptom-free period, the patient was admitted to the emergency unit on March 31, 2020, due to several symptoms including fever, dizziness, cough and nausea. About 5 days later, on 5 June 2020, he tested positive with a genetically different variant from the initial test and also presented a more severe form of the disease by being hypoxic and requiring oxygen administration. Sequencing data showed that although two samples from two positive tests belonged to 20°C clade, they each contained distinct single nucleotide variants that were not present in the other [23].

In South America, the first confirmed reinfection case was reported in Ecuador. The history of a 46-year-old male on 12 May 2020, showed headache and drowsiness from the past 3 days. Antibody assay as a rapid antibody test resulted in positive for IgM but negative for IgG. He tested positive using RT-PCR on 20 May 2020; however, he did not require hospital admission and his symptoms resolved a few days later. On 20 July 2020, he became symptomatic again with more severe manifestations including dyspnea, productive cough and fever of 38.5°C. RT-PCR results on 22 July 2020, revealed positive for the infection and after signs of improvement, he was negative for RT-PCR following several days. Also, seroconversion of anti-Nucleocapsid IgG and positive result for IgM by ELISA occurred on 18 July 2020. Genome sequencing of the sample showed that the initial event was caused by 20A clade and the second one by 19B clade without any common mutations. These data are suggestive of reinfection by a new variant rather than relapse of infection [24].

Similar cases from India in terms of reinfection were analyzed by the case study of Gupta *et al.* [25]. The cases were two healthcare workers, a 25-year-old man and a 28-year-old woman who first tested positive using RT-PCR on 5 and 17 May 2020, each. Despite being asymptomatic they were admitted to the hospital and after discharge, they both contracted with novel clade and retested positive on 21 August 2020 and 5 September 2020, respectively. Similar to the first episode they were both symptom free. The lower cycle threshold (C_t_) for RT-PCR in the second infection indicated a higher viral load. Interval of more than 3 months between episodes, detection of novel mutations especially S:N440K in the woman victim and higher viral load in the second infection highly contribute to the occurrence of reinfection [25].

Reinfection with different clade is also documented in the case that occurred in Belgium. A 51-year-old female with no immune deficiency was found symptomatic with headache, fever, chest pain, coughing and dyspnea, and tested positive with RT-PCR on 9 March 2020. After 3 months of the first event, she developed milder symptoms, and on 10 June 2020 results of RT-PCR with anti-Nucleocapsid IgG 1-week later were indicative of infection by distinct viral clade. Genome sequencing results unveiled that the initial infection was due to B.1.1 lineage and the second one related to clade A and two clades differed at 11 mutations across them IgG seroconversion and span of over 3 months between two episodes, is consistent with the occurrence of reinfection [26]. Characteristics of reinfected cases are summarized in Table 1.

**Table 1.** Characteristics of reinfected cases.

Country	Patient age (years)	Patient sex	First episode variant	Symptoms of the first episode	Interval between first and second infection (days)	Second episode variant	Symptoms of the second episode	Ref.
China	33	Male	B.2	Cough, fever and sore throat	142	B.1.79	Asymptomatic	[22]
USA	25	Male	20C	Symptomatic	70	20C but different SNPs was existed from the first infection	More severe symptoms	[23]
South America	46	Male	20A	Headache, drowsiness	62	19B	More severe manifestation including dyspnea, productive cough and fever	[24]
India	25	Male	Not mentioned	Asymptomatic	104	Not mentioned	Asymptomatic	[25]
India	28	Female	Not mentioned	Asymptomatic	108	Probably Delta which contains S:N440K mutation	Asymptomatic	[25]
Belgium	51	Female	B.1.1	Headache, fever, chest pain, coughing and dyspnea	Over 90	A	Milder symptoms	[26]

SNP: Single-nucleotide polymorphism.

### Short immunity window as a risk factor for reinfection

Since the reinfection phenomenon is common among coronaviruses family [8], there is an unquestionable need to explore the immunity period provided by SARS-CoV-2 infection. The main antibody responses against SARS-CoV-2 are contributed to Spike and Nucleocapsid. Compared with mild and moderate infection, a severe form of the disease is associated with higher antibody response and antigen binding capacity [27]. However, the presence of IgG cannot protect from reinfection [28].

In the comprehensive cohort study of Gaebler *et al.* [29], they revealed that despite a remarkable decline in neutralizing activity of anti receptor binding domain (RBD) and anti-Nucleocapsid antibodies, the levels of antibodies remained above the detection threshold after 6 months. Antibodies with the greatest dip in titers were from IgM (53%) and IgG (32%) classes. However, they found that during 6.2 months of follow-up, memory B cells evolved and contributed to the production of more robust antibodies capable of neutralizing mutant RBDs [29].

Another large analysis by Dan *et al.* [30] from 188 COVID-19 subjects demonstrated that during 8 months after infection, the most stable antibodies were anti-Spike and anti-RBD IgA. Anti-Spike IgG also retained its stability compared with other antibodies with a half-life of 140 days. In assessing memory B-cell level modifications, they found that both RBD and Spike-specific memory B cells underwent a clonal expansion and increased in frequency in the first 4–5 months. Antibody expression of B cells against Nucleocapsid, Spike, and RBD resulted in short-lived IgM but a steady IgG and IgA for more than 6 months. Among the T-cell population, CD4^+^ memory cells developed in the majority of subjects (93%) in the first 1 month of infection and retained their level after 6–8 months, in contrast, CD8^+^ memory cells were impacted by a significant decrease in frequency by 6–8 months timeframe [30].

Despite diminishing immunity in individuals with a healthy immune system that may act as a predisposing factor to reinfection, in the study of Pal *et al.* having uncontrolled diabetes also might be a risk factor not only for infection relapse but also for reinfection because of deteriorated adaptive immune responses in various aspects [31].

Overall, the immunity window against SARS-CoV-2 appears to wane after several months of infection and the longer the period, the more it favors reinfection. However, as the exact protective period provided by anti-SARS-CoV-2 antibodies is still unclear and yet to be determined [9], these data clarify the necessity of considerations in vaccine development.

### Rates of reinfection & acquired protection

Even though several cases of reinfection documented around the world, the occurrence of reinfection remains to be unknown despite some discrepancies in reports from different populations [32].

Studies also demonstrated the effect of previous exposure as well as vaccination in protecting from reinfection. A study from five countries reported that former exposure reduced the rate and severity of reinfection and this protection increased by receiving adjuvant protein COVID-19 vaccine candidate S-Trimer SCB-2019 vaccine [33]. It should be mentioned that this protection varies in different vaccines and variants. For example, the reinfection rate of Omicron is three-times more than previous variants [34].

These findings may imply that a large proportion of individuals who contracted the virus are still protected against second infection [35]. However, seeking the incidence of reinfection is vital for further concerns relating to vaccine-acquired immunity and health policies among societies. A large study from Denmark reported a protection rate of 77–83% against the second infection. During the second spike in cases from 1 September to 1 December 2020, 0.65% of previously RT-PCR-positive subjects retested positive. The interval between two surges was more than 3 months thus, reinfection better fits the situation [36].

Investigations in Austria also confirm the notion of high protection after initial infection. In this analysis, the odds ratio evaluated for infection in formerly infected subjects versus the general population was 0.09 (95% CI: 0.07–0.13) and a subsequent reinfection rate of 0.27%, meaning prior infection could protect individuals to the extents current vaccines provide immunity [37]. In a UK study by Breathnach *et al.* [38] which spanned for 8 months, among 10,727 subjects with previous positive PCR results, eight cases (0.07%) were identified as positive again in the second wave from 1 August to 30 December 2020 (more than 60 days interval according to the latest data), versus 713 positives (1.29%) in 55,274 subjects with no prior infection. The relative risk of developing the infection in previously infected individuals in the second wave was 0.0578 (95% CI: 0.0288–0.1160). It was also noted that the reinfection rate was 1.69% in December with no evidence of reinfection in the past 7 months of study [17,38]. This is in line with other studies suggesting a substantially lower risk of infection among individuals with prior history of infection [35,39].

A broad cohort study of reinfection incidence in UK healthcare workers unveiled that among healthcare workers with anti Spike IgG-positive results, the rate of PCR positivity was 0.12 (95% CI: 0.03–0.47; p = 0.002) considering the first and second surge of the disease in the UK with more than 3 months apart [39]. Similar work by Hall *et al.* exhibited lower odds of reinfection by at least 75% in previously infected subjects [40]. A report from Qatar was also indicative of the rare occurrence of reinfection with the rate of 0.02% (95% CI: .01–.02%) [41]. In one full-scale systematic review conducted by Murchu *et al.* [42], while reporting the data from the Denmark cohort study, the adjusted relative risk of reinfection among individuals aged above 65 years was reported as 0.53 (95% CI: 0.37–75), whereas for other younger age groups including 0–34, 35–49 and 50–64 were 0.17,0.20 and 0.19, respectively. This might be regarded as a more competent immune system in the younger population in case of reinfection with SARS-CoV-2; however, more evidence is required on this subject [36,42].

## Reactivation

### Review of cases

Evidence of viral persistence rather than reinfection in other coronaviruses is reported in the forms of prolonged RNA shedding [43,44]. Contrary to SARS-CoV-2 reinfection, recurrence or relapse of disease is relatively prevalent [45]. This may partly be due to factors leading to re-positivity of PCR tests ranging from false negative PCR results at the time of discharge to the formation of dormant viral particles after the symptom resolution [46]. Here, we will discuss some documented cases of reactivation in addition to its prevalence.

A case report from China presented the history of a 46-year-old woman experiencing fever on 17 January 2020, with recent travel history to Wuhan, China. Following imaging with high-resolution computed tomography (CT) favoring infection with SARS-CoV-2, she tested positive for RT-PCR on 24 January 2020. The patient’s symptoms improved after receiving therapies along with lesion resolution appeared in HCRT. Also, RT-PCR tests performed on 28 and 30 January 2020 were reported negative until 2 February, when the result turned positive. Post discharge follow-ups exhibited negative RT-PCR results until 18 February 2020. According to the report, false negative results due to the sample retrieval procedure may lead to the re-positivity of the test on 2 February 2020 [4].

Relating COVID-19 reactivation in individuals with underlying health conditions including malignancies, there is a report of a 55-year-old woman who suffered from coronary artery disease, diabetes mellitus and also B-cell acute lymphocytic leukemia while receiving chemotherapy. On 8 April 2020, she tested positive using PCR due to symptoms of fever and abdominal pain. During the progression of the disease, her inflammatory markers were elevated and after 18 days of infection, she was discharged with two negative PCR tests on 7 and 11 May 2020. On 18 June 2020, she developed severe symptoms including high fever (40.3°C), abdominal pain and bloody diarrhea and PCR test showed positive this time, while her antibodies against COVID-19 depleted thoroughly. The severe form of disease made her admitted to ICU; however, she was discharged from the hospital after several days. The explanation for the positive result of PCR for the second time relies on residual viral shedding. Moreover, incomplete viral clearance due to administration of immunosuppressive medications particularly lymphocyte and antibody depleting agents may result in viral reactivation [32].

There is also evidence of SARS-CoV-2 recurrence in children as elucidated in the case report of Yoo *et al.* [47]. The 8-year-old boy presented with a cough from the past 3 days confirmed with COVID-19 on 3 March 2020. During hospital admission, his symptoms improved until the qRT-PCR result turned negative on day 14 of admission. He developed progressive symptoms with mild fever, 4 days after hospital discharge and on 4 April 2020, a qRT-PCR test was performed and resulted positive again despite other laboratory and imaging findings being inconclusive. During the relapse, his overall condition was reported to be good. Serial qRT-PCR tests remained positive until 11 and 12 April 2020 when two qRT-PCR-negative results were indicative of infection resolution and no hospital stay is required [47].

Another patient related to reactivation was a 40-year-old man suspected of SARS-CoV-2 infection on 18 January 2020. Before the PCR test, he underwent CT imaging and lesions were indicative of pneumonia. On 23 January 2020, his PCR results confirmed COVID-19. His symptoms became deteriorating quickly so he ended up using a ventilator. However, after administration of methylprednisolone and i.v. antibodies, his symptoms improved and discharged from the hospital on 4 and 6 February 2020 with two negative PCR results. Only about 5 days later, he became symptomatic with fever and on 14 February 2020, his PCR results turned positive for the second time, and was admitted to the hospital. The patient recovered quickly and was discharged with two subsequent negative PCR tests on 1 March 2020, along with imaging findings improvement. Lab data revealed that inflammatory markers of LDH, ferritin and IL2R were highly elevated during the second episode with LDH being mainly high from the onset of the infection. Serological assays also showed a decline in both IgG and IgM levels against SARS-CoV-2. In addition, genomic data unveiled that he carried a mutation that is associated with B-cell immunodeficiency. Factors that made this patient prone to relapse of infection may contribute to a lack of antibodies which causes improper viral clearance. Also, the possibility of false negative PCR results at the time of discharge in the first episode was noted in the report [48].

A study from India reported a male with five-times reactivation during 7 months, who eventually died. Each time the RT-PCR result was positive and had more symptoms than the previous time. He never developed antibodies. Finally, a biopsy was done and thymoma was diagnosed. As he did not have contact with SARS-CoV-2-infected patients and the interval between admissions was less than 2 months, he was considered as a reactivation [49].

Characteristics of reactivated cases are summarized in Table 2.

**Table 2.** Characteristics of reactivated cases.

Country	Age (years)	Sex	Underlying disease	Possible explanation	Ref.
China	46	Female	Not reported	False test results	[4]
USA	55	Female	Malignancy (B-ALL) Diabetes mellitus Coronary artery disease	1. Residual viral shedding 2. Incomplete viral clearance due to administration of immunosuppressive medications	[32]
Korea	8	Male	Not reported	Detection of viral RNA does not necessarily mean that infectious viruses are shedding	[47]
China	40	Male	B-cell immunodeficiency	Mutations in *TRNT1* gene	[48]
India	41	Male	Thymoma	No antibody development	[49]

B-ALL: B-cell acute lymphocytic leukemia.

### Reactivation or SARS-CoV-2 re-positive test prevalence

As a myriad of COVID-19 reactivation cases declared from all around the globe with also being relatively common phenomenon [45,50], here we will discuss prevalence studies to figure out this recurrent infection or positive test due to RNA fragments shedding since they are not always equivocal and discrimination between these two concepts would be difficult [18,50].

An all-inclusive systematic review and meta-analysis conducted by Ren *et al.* [51], demonstrated that the rate of PCR re-positive results in discharged patients was roughly 12% with high variability in patients’ risk factors leading to recurrence of positive results [51]. Another study by Dao *et al.* [45], reported recurrence of the positive test in discharged patients by RT-PCR in a broad range from 2.4 to 69.2% by various causes with the majority of the patients experiencing mild to moderate symptoms [45].

In the analysis carried out in Italy on 1146 discharged patients with COVID-19, 125 (10.9%) patients reported having relapse of the infection in asymptomatic form detected by positive RT-PCR test, suggesting recurrence of the infection is a rather frequent [50]. These findings are in line with the largest cohort conducted by Bongiovanni *et al.* [50] that displayed a recurrence rate of RT-PCR tests in discharged patients by more than 10% although, they were mainly asymptomatic [50].

A retrospective study from China on 55 patients, analyzed that five out of 55 (9%) patients experienced reactivation of infection with the majority bearing moderate symptoms including fever, sore throat and fatigue. However, no severe case of disease or death was reported in the study [52]. The pieces of evidence favoring the prevalence of recurrent infection and PCR re-positivity are also reflected in the study of Huang *et al.* [53]. This study not only evaluated rates regarding both infection recurrence and PCR test positivity for a second time but also proposed a prediction model for developing recurrent SARS-CoV-2 positivity by embodying 18 clinical factors. In terms of recurrent PCR positivity, they documented the rate of 16.7% with the majority being younger than the control group and admitted with mild symptoms [53].

### Predictive markers of reactivation

Concerning COVID-19 recurrence prediction, several risk factors contribute to the susceptibility of the patients against the second hit of the infection. The notable risk factors were categorized in the study of Ye *et al.* [52] into immunity status of the patients alongside underlying diseases accounting for host status, virological factors including viral load and the level of immunosuppression caused by therapies [52]. Similar predisposing agents were introduced in the study of Zhou *et al.* [54].

While investigating more in-depth, Chen *et al.* [55] found an association between potential risk factors and recurrence of SARS-CoV-2 RNA tests positivity after analyzing the sera samples and imaging features on 1087 COVID-19 patients retrospectively. These factors include raised serum IL-6 level, elevated lymphocyte count (>1.1 * 10^8^/l) and CT images showing a consolidation [55]. Additionally, another study concluded that among liver transaminases, AST and ALT may be the significant predictive factors for recurrent infection since their serum level may be elevated in the course of SARS-CoV-2 infection [56,57].

### Possible explanations for SARS-CoV-2 reactivation or recurrent positive test

Current evidence for possible causes of true recurrent infection or test positivity are mainly divided into four areas: issues with sample retrieval procedure and tests sensitivity; prolonged viral RNA shedding; incomplete viral clearance and activation of viral exosomes; testing at the improper time with a long interval between negative test and discharge [46,58]. Sample-related issues may occur as the higher viral load in the lower respiratory tract than at the site of sampling (upper tract) due to viral tendency and higher expression of ACE2 receptor in the lower tract, the technique used by an operator in sampling and various errors in handling specimen before testing [4,18,48,50]. In addition, due to the relatively low sensitivity of PCR tests with a daily gradual decline after symptom onset for identifying SARS-CoV-2 RNA, many negative results at the discharge could be considered false negatives [45,59–61].

The concept of residual RNA shedding in patients with COVID-19 is addressed in several studies. Notably, persistent RNA detection or prolonged nucleic acid conversion time does not imply relapse or recurrence of infection and is also not associated with viral transmissibility or infectivity [45,62–64]. In the study of Li *et al.* (2020), viral RNA shedding appeared not to be a rare incident by measuring the shedding median duration of 53.5 days after symptom onset. It was also reported that patients with severe illness had a longer viral RNA shedding compared to patients with mild to moderate symptoms [41]. These data are confirmed in the studies of Xu *et al.* [65] and Noh *et al.* [66] with the median viral shedding duration of 17 and 24.5 days, respectively [65,66].

In addition to the use of immunosuppressive agents and comorbidities [61], the role of dormant viral exosomes in the incomplete clearance of the virus is noteworthy. In the study of Elrashdy *et al.* [46], it is indicated that similar to SARS-CoV, SARS-CoV-2 could induce forming of double-membrane vesicles or exosomes filled with a large number of viral particles. This process is associated with the rough endoplasmic reticulum of the host cell. Using double membrane vesicles or exosomes the virus can hide in the cell as a trojan horse strategy to begin a new infection after being secreted during exocytosis at the proper time. It was also noted that exosomes could transport ACE2 receptors from host cells to the recipient cells. Together, exosomes may play a crucial role in SARS-CoV-2 viral dispersion through extracellular transport of viral particles [46].

## Conclusion

SARS-CoV-2 along with its growing pandemic has been challenging healthcare systems around the world while leaving many questions unanswered regarding unknown aspects of the virus. The occurrence of reinfection or reactivation of the virus is one of the mysterious concepts of viral immunity and also raises concerns about vaccine-acquired protection. Reinfection is defined as an infection by a distinct viral clade rather than the first episode of infection with its own definite criteria [16]. On the other side, reactivation or relapse of the infection takes place after symptom resolution whereas the virus is still present but is regarded as dormant and may become active again. Reactivation also harbors its marker by ruling out possibilities for reinfection. Even though one must distinguish the true reinfection from reactivation, the distinction between reactivation and tests re-positivity is also notable since they are not equal [50]. Detecting the viral RNA using the RT-PCR method is not translated to active infection or being contagious [45,62–64] since prolonged RNA shedding in COVID-19 patients which consists of residual viral RNA or dead viral particles is not a rare occurrence [41]. Additionally, false negativity and low sensitivity of these tests are attributed to negative results at discharge in patients with unresolved infection thus, recognized as retested positive in the following days of the disease [45,59–61].

We reviewed cases of both reinfection and reactivation from various regions and from that reinfection appeared to be a rare phenomenon with a rate of mainly below 1% [36–39,41]. In contrast, reactivation rates were suggestive of being a common incident [45,50]. While exploring the immunity period conferred by SARS-CoV-2 infection, data were in favor of short-lived protection that may not extend beyond 6–8 months after infection [29,30]. Therefore, it unveils the possible explanation for reinfection and also milder symptoms in previously infected individuals [35]. Finally, we investigated predictive markers plus factors leading to reactivation or positive test results after the first infection. A distinguished mechanism for viral dissemination in recurrence of the infection was introduced in which viral particles remain dormant in exosomes and are transferred to other cells in case of reactivation [46].

The emergence of reinfection and reactivation depicted a long path to containing the pandemic. The appeared challenges are also crucial for vaccine development and health policies regulated in societies to combat the spread of the virus. Further studies are required on immunity window and in-depth analysis of virus–host interaction to shed light on hidden features of the virus.

## Future perspective

Due to the epidemic of SARS-CoV-2, it is necessary to study this virus and its diseases and its different dimensions of it as much as possible in order to prevent and treat COVID-19 more effectively. Therefore, being aware of the possibility of reinfection or reactivation of this virus will help us to identify and follow-up the individual at risk of recurrence, and thus take better care of the health of the patient, family and community. For example, people with a genetic defect, immune system disorders or malignancies are more prone to reactivation; therefore, medical staff should be alert to the reactivation of the virus in these people.

On the other hand, according to the data obtained from the articles, the rate of reinfection is very low, while in practice, we see a high number of people in the community who have been infected with COVID-19 several times, and this is especially the case in the new waves which could be due to the emergence of new variants and new mutations in the virus in addition to diminishing protection conferred by antibodies, especially IgG. Therefore, it is suggested that more studies be done on reinfection by examining a large number of people.

**Executive summary**

**Reinfection**

- Reinfection occurs when an individual becomes infected again after recovering from a previous infection.
- The virus is from a different clade compared with the previous infection.
- There is often an episode interval of at least 2–3 months.
- The symptoms of different episodes may be completely different.

**Reactivation**

- Reactivation deals with the process of viral activation after an episode of infection and its recovery.
- Reactivation occurs due to several factors including inborn errors of the immune system, or a decrease in immune function caused by malignancy or the use of immunosuppressive drugs and or chemotherapy.
- Reactivation could be explained by the presence of exosomes containing the virus or viral particles.
- To confirm reactivation, the viral clade of both episodes should be of the same clade.
- Reactivation predominantly occurs within 60 days of the infection with the most common interval of 10–27 days.
- It is crucial to discriminate persistent shedding of virus from reactivation by at least two negative RT-PCR results.
- Reactivation is a common phenomenon especially in healthy people without underlying disease or immune system disorders.
